# Supplementary material for: Hamstring Strain Injury Patterns in Spanish Professional Male Football (Soccer): A Systematic Video Analysis of 78 Match Injuries
Source: J Funct Morphol Kinesiol. 2025 May 31;10(2):201. doi: 10.3390/jfmk10020201 (PMC12193792; doi:10.3390/jfmk10020201)
Supplement: Supplementary file 1 [file jfmk-10-00201-s001.zip › jfmk-3592205-supplementary.pdf]

Journal of
Functional Morphology and Kinesiology

Supplementary Materials

Supplementary Materials S1

Table S1: Description of the different criteria and categories used to analyze the videos.

1. Contextualization

1.1. Contact

1.1.1. Contact injury: is defined as any injury sustained through the influence of contact with an opponent.

1.1.2. Non-contact injury: is defined as any injury occurring without contact with an opponent.

1.2. Specific position

1.2.1. Goalkeeper

1.2.2. Fullback: it will be considered FB when the team plays with a 4-defense system.

1.2.3. Central defender

1.2.4. Wingback: it will be considered WB when the team plays with a 5 or 3 defense system.

1.2.5. Central midfielder

1.2.6. Wingers/Wide midfielders

1.2.7. Attacking midfielder

1.2.8. Striker

1.2.9. Second striker

1.3. Ball

The *ball* criterion takes into account whether the presence of the ball is relevant and affects the moment of the injury, conditioning the player's actions at that moment.

1.3.1. Yes

1.3.2. No

1.4. Time of injury relative to the game (min)

1.4.1. First half

1.4.1.1. 0-15 beginning

1.4.1.2. 16-30 medium

1.4.1.3. 31-45 final

1.4.2. Second half

1.4.2.1. 46-60 beginning

1.4.2.2. 61-75 medium

1.4.2.3. 76-90 final

1.5. Situation

1.5.1. Offense: the *offensive* situation is considered when the injured player's team has possession of the ball and the initiative of the game.

1.5.2. Defensive: the *defensive* situation is considered when the player of the injured team that does not have possession of the ball tries to take it away and prevent the opponent's progress.

2. Injury analysis

2.1. Injury pattern

2.1.1. Sprinting-related movement pattern: the injury must occur during a dynamic action in which the player is running at high speed or sprinting.

2.1.2. Stretch-related movement pattern: the injury must occur starting from a static or low-speed movement situation in which the player performs a hip flexion together with a knee extension, generating an overstretching of the hamstring musculature.

2.1.2.1. Open kinetic chain: the injury occurs during a movement that presents a hip flexion together with a knee extension, causing an overstretching of the hamstring musculature, but when the injured leg is moving without touching the ground (e.g., ball strikes, controls...).

2.1.2.2. Closed kinetic chain: the injury occurs during a movement that presents a hip flexion together with a knee extension, causing an overstretching of the hamstring musculature, but when the injured leg touches the ground, usually in braking and stopping actions (e.g., braking, landing...).

2.1.3. Combined movement pattern 1: will be of this type when there is a combination of the sprinting pattern together with the open kinetic chain stretching pattern.

2.1.4. Combined movement pattern 2: will be of this type when there is a combination of the sprint pattern together with the closed kinetic chain stretch pattern.

2.2. Trajectory

2.2.1. Linear

2.2.2. Curvilinear

2.3. Technical action

2.3.1. Dispute: the injury occurs during a dispute when, normally, two players are fighting for the ball and possession of the ball is not defined.

2.3.2. Shooting: the injury occurs when the player takes a shot or kicks the ball in the direction of the goal.

2.3.3. Pass: the injury occurs when the player gives the ball to a teammate intentionally (usually well oriented towards the teammate receiving the pass). All types of passes are included in this category: short, long, low, lifted, volley...

2.3.4. Cross: the injury occurs when the player hits the ball to send it into the opponent's area from a lateral zone with the purpose of creating a situation for a teammate to shoot. If the strike is inside the area, it is not considered a cross, but a pass. This category includes all kinds of different crosses: short, long, low, lifted, volley...

2.3.5. Clearance: the injury is considered a clearance-type injury when the player is injured while moving the ball away from the goal to resolve a compromising situation near his goal.

2.3.6. Control: the injury occurs when the player tries to dominate the ball, it can occur in offensive actions such as trying to control the ball after a teammate's pass, or in defensive actions such as trying to snatch the pass from an opponent.

2.3.7. Driving: the injury occurs when the player performs an intense run transferring the ball without passing it to a teammate.

2.3.8. Tackling: the injury occurs when the player tries to impede the progress of the opposing player in possession of the ball by throwing himself to the ground and attempting to stop the progress.

2.3.9. Steal: the player being analyzed will be injured when he tries to regain possession of the ball by taking it away from the opponent.

2.3.10. Ø (none/stopped)

Table S2. Examples of illustrations of the different injury patterns analyzed.

	**Assumed Injury Frame**
**A**	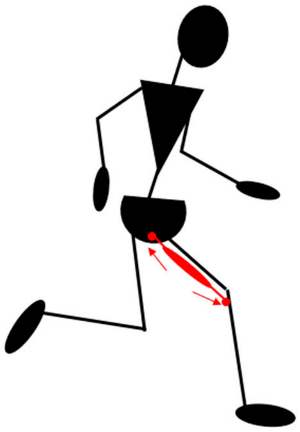

**B**	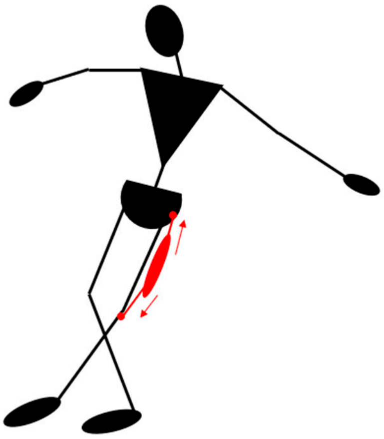
**C**	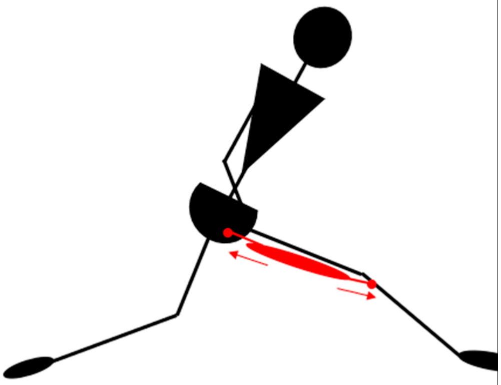 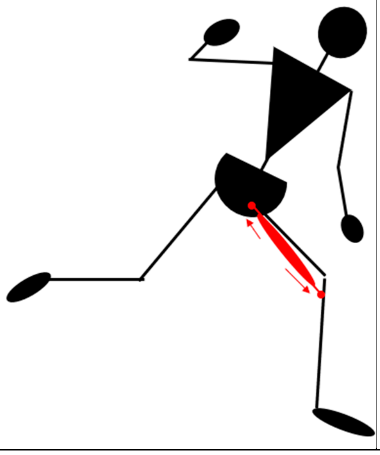 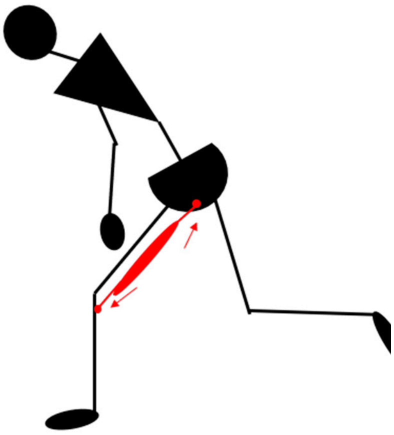
**D**	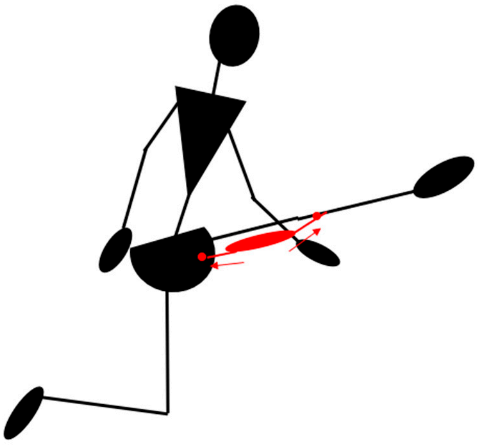

**E**	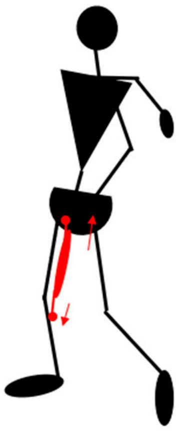	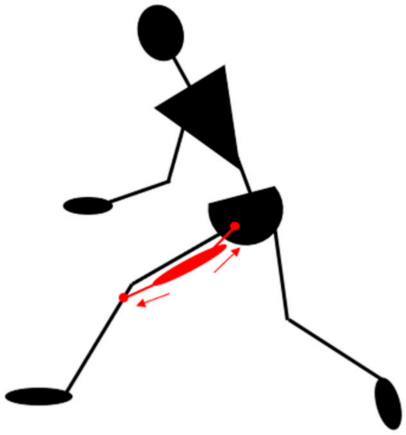	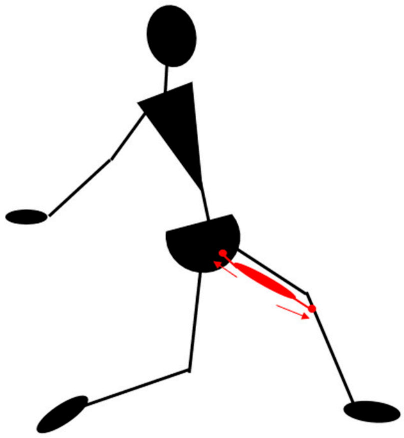

A) Sprint-type pattern, B) ST-OC pattern, C) different sub-patterns of the ST-CC pattern, D) COMB1 pattern, and E) different sub-patterns of the COMB2 pattern.
